# Are Adolescents with ADHD Interested in Genetic Testing for Nicotine Addiction Susceptibility?

**DOI:** 10.3390/ijerph7041694

**Published:** 2010-04-14

**Authors:** Linda J. Herbert, Leslie R. Walker, McKane E. Sharff, Anisha A. Abraham, Kenneth P. Tercyak

**Affiliations:** 1 University of Maryland Baltimore County, 1000 Hilltop Circle, Baltimore, MD 21250, USA; E-Mail: jones5@umbc.edu; 2 Seattle Children’s Hospital, 4800 Sand Point Way NE, Seattle, WA 98105, USA; E-Mail: leslie.walker@seattlechildrens.org; 3 Georgetown University Medical Center, 3300 Whitehaven Street, NW, Washington, DC 20007, USA; E-Mails: mes225@georgetown.edu (M.E.S.); aa359@georgetown.edu (A.A.A.)

**Keywords:** attention-deficit/hyperactivity disorder (ADHD), genetic testing, nicotine addiction

## Abstract

It has been well-established that some adolescents diagnosed with attention-deficit/hyperactivity disorder (ADHD) are at increased risk for cigarette smoking. Current research on the genetic basis of this association could ultimately translate into genetic tests capable of identifying smoking-prone adolescents with ADHD. In this study we examined 81 ADHD affected adolescents’ (age 13–21) interest in genetic testing for nicotine addiction susceptibility. Fifty-seven percent of adolescents indicated a fair amount of interest or more in testing. Most adolescents indicated that the personal information revealed from testing would be either useful (29%) or interesting (37%). Implications for genetically-informed smoking prevention and cessation interventions in high risk adolescents with ADHD are discussed.

## 1. Introduction

According to the National Survey on Drug Use and Health, approximately 27.6% of the U.S. population aged 12 years and older engages in tobacco use at least once per month [1]. Over three million of these users are minors—or roughly 12% of America’s adolescents [1]. Cigarettes are the most common form of tobacco use, and roughly 20% of teenagers currently smoke cigarettes [2]. Given the consequences of smoking, tobacco use is a major public health concern. It remains the leading cause of preventable deaths, responsible for at least 400,000 deaths annually, and is estimated to cost $82 million/year in lost productivity [3]. Most adult smokers began smoking when they were adolescents [4]. Thus, it is crucial to identify risk factors for smoking, and provide education, counseling, and interventions to prevent and/or stop smoking in adolescents who are most at risk [5].

One group of adolescents that has been identified to be at high risk for smoking is adolescents diagnosed with attention-deficit/hyperactivity disorder (ADHD) [6,7]. ADHD is a neurobehavioral syndrome that begins during childhood and affects approximately 3–5% of children and adolescents in the U.S. [8]. ADHD is characterized by symptoms of inattention and/or hyperactivity-impulsivity and impairment in the areas of home, school, and/or social functioning [8]. The increased risk for adolescents with ADHD to become smokers has been well-established through several studies. According to Lambert and Hartsough [9], by age 17 approximately 47% of adolescents diagnosed with ADHD report daily smoking as compared to 24% of adolescents without a diagnosis of ADHD. In another study, adolescents who display clinically significant symptoms of inattention, a major symptom of ADHD, are almost three times as likely as healthy adolescents to be current smokers [10].

Though the cause for this association remains unknown, it has been hypothesized that symptoms of ADHD, such as impulsivity, predispose adolescents to engage in a number of risky behaviors, including smoking [11]. Concomitant symptoms of ADHD, such as inattention, are also proposed to maintain smoking behavior due to the fact that nicotine has remediating/psychostimulating effects on attention, arousal, and concentration [6,12]. Indeed, preliminary studies with adults suggest that individuals who have been diagnosed with ADHD have less success in smoking cessation programs than adults without ADHD [13]. Despite this evidence, the only known smoking prevention program directly tailored to adolescents with ADHD was pharmacologically-based and proved ineffective [14]. At this time there is a need for more advanced (*i.e.*, targeted and tailored) smoking interventions that address the biological, psychological, and social aspects of smoking initiation and cessation [15].

Numerous twin studies demonstrate that approximately 60%–70% of the variance in nicotine addiction may be attributable to inherited factors [16,17]. Thus, a burgeoning area of research has focused on both candidate gene approaches and genome-wide association study (GWAS) approaches to provide a more comprehensive perspective on the biological propensity for nicotine addiction. Briefly, candidate gene studies examine single genes that are hypothesized to influence the development of health concerns by contributing to a particular disease phenotype [18]. Several candidate genes have been proposed to be linked to smoking uptake and nicotine dependence. The impact of the cytochrome P450 *CYP2A6* gene has gained prominence in the literature due to extensive replication studies [19,20]. Individuals with the wild genotype of *CYP2A6* tend to metabolize nicotine faster, are more likely to be dependent on nicotine, and are more likely to relapse after an attempt to quit smoking [21]. Other genes shown to be associated with earlier smoking initiation and nicotine dependence include genes that code for nicotinic acetylcholine receptor (nACHr) subunits, the dopamine transporter gene (*SLC6A3*), the dopamine D3 and D4 receptor genes (*DRD3*, *DRD4*), and polymorphisms in a nicotine metabolizing enzyme gene *CYP2A6* [21].

Whereas candidate gene studies focus on specific genes, GWAS examine the genome as a whole to assess the impact of multiple single nucleotide polymorphisms (SNPs) on health outcomes [22,23]. GWAS are usually exploratory in nature and advantageous in tobacco control research because they can provide information about previously studied genes and SNPs as well as novel genes and SNPs that have not yet been identified [22]. Furthermore, GWAS have the potential to clarify the interaction of genetic and environmental influences, such as low socioeconomic status, peer smoking, and maternal smoking, on smoking behavior [18]. Several GWAS have identified SNPs that may be related to cigarette smoking behaviors. Although a complete review of those works is well-beyond the scope of this article, several intriguing findings are noteworthy. For example, there is preliminary evidence that polymorphisms in the *CHRNA3-CHRNA5* nicotinic receptor subunit gene cluster are related to cigarette use per day [24], and in the gene *NRXN1* are related to nicotine dependence [25,26]. SNPs that play a role in glutamate (a neurotransmitter) signaling, such as in genes *GRIN2B*, *GRIN2A*, *GRIK2*, and *GRM8*, are elevated after nicotine administration, suggesting they may affect vulnerability to smoking initiation and maintenance [27]. Specific SNPs have also been associated with success in stopping smoking, including those in genes *ATP1BL1*, *CSMD1*, *DDX10*, *DSCAM*, *FLJ22746*, *RPL21P12*, and *SLC9A9* [27–30]. Studies indicate that the SNPs/variants for *CNTN6*, *CSMD1*, *LRRN6C*, and *SEMA3C* are associated with vulnerability to substance addiction as well [31,32].

One outgrowth of these discoveries may be the advent of predictive genetic tests of nicotine addiction susceptibility [33]. Genetic testing for nicotine addiction susceptibility would allow health care providers to identify those adolescents at greatest risk for smoking [34,35]. Given the high rate of smoking among adolescents with ADHD, genetic testing may offer an additional strategy to reduce smoking risk by motivating adolescent behavior change [36]. Genetic information could help target adolescents with ADHD who are at high risk for smoking and enable health care providers to tailor interventions to address both the adolescent’s genetic and neurobehavioral predispositions to smoke (*i.e.*, by inhibiting genes that make smoking more likely and addressing symptoms of ADHD such as impulsivity) [37,38]. Adolescents who are knowledgeable about their genetic nicotine addiction susceptibility may be more motivated to refrain from smoking or make an effort to stop smoking. Physicians support the notion that knowledge of personal nicotine addiction susceptibility could motivate patients to quit smoking [39] and indicate interest in using genetic testing to match patients to optimized smoking cessation strategies [40]. Pharmacogenetic studies have begun elucidating which persons may have the most success with nicotine replacement treatments for stopping smoking [41], and this promising approach could be adapted for primary prevention as well.

Preliminary evidence suggests adolescents are interested in learning about their genetic risk for a wide range of health conditions. Studies on genetic testing for several health concerns, including adult-onset breast cancer, Tay-Sachs disease, hypercholesterolemia, osteoporosis, hemachromatosis, heart disease, and nicotine addiction, show that a majority of adolescents are interested in receiving genetic information regarding their personal risk [36,42–45]. These studies indicate that adolescents with a family member who has the target condition (such as breast cancer), or who are members of ethnic groups with a higher risk of a disorder (such as Tay-Sachs disease), and/or who perceive the condition to be severe tend to be most interested in genetic testing [43].

With respect to nicotine addiction susceptibility testing, Tercyak and colleagues [36] found that approximately 62% of general population adolescents attending routine medical appointments would be interested in this form of genetic testing if it became available. Interest tended to be associated with higher socioeconomic status and better school performance. When asked to clarify reasons for their level of interest, 30% of adolescents wanted the information because it would directly affect their efforts to prevent or stop smoking. This suggests that nicotine addiction susceptibility test results may be a motivator for adolescents to alter their smoking intentions and practices. However, although this study provided significant information regarding the attitudes of adolescents in the general population, the sample did not include adolescents diagnosed with ADHD. It is crucial to assess the attitudes of adolescents with ADHD towards nicotine addiction susceptibility testing in particular because they are at increased smoking risk.

The purpose of the present study was to determine the interest of adolescents with ADHD in nicotine addiction susceptibility testing and to clarify adolescents’ reasons for their level of interest in such testing. It also sought to identify if adolescent smoking behavior was positively related to interest. Consistent with the results of the Tercyak and colleagues’ study [36], we hypothesized that the majority of adolescents with ADHD would be interested in this form of predictive genetic testing. We approached this issue by sampling affected adolescents presenting for clinical care of their ADHD symptoms, and administering a self-reported behavioral assessment of key constructs of interest (see Experimental Section for complete details). Data were analyzed descriptively by employing univariate statistical procedures, followed by a bivariate analysis of the relationship between adolescent smoking behavior and interest in genetic testing.

## 2. Results and Discussion

### 2.1. Interest in Genetic Testing

**Figure 1.**
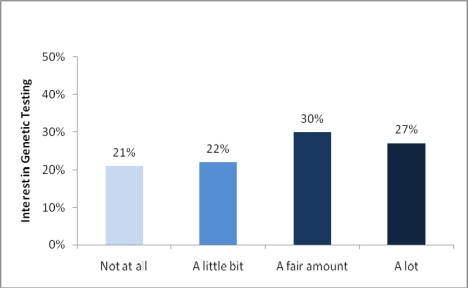
Adolescents’ interest in nicotine addiction susceptibility testing (*n* = 87).

Most adolescents in this sample expressed interest in genetic testing (see Figure 1). Fifty-seven percent of the sample indicated a fair amount of interest or more.

### 2.2. Reasons for Interest in Genetic Testing

The reasons that adolescents provided for their level of interest in genetic testing were classified into five categories based on their indication that participation in testing would be: (1) useful, as it would likely affect their smoking decision making and smoking behavior, (2) generally interesting, but unlikely to impact upon decision making about smoking or smoking behavior, (3) irrelevant, (4) unimportant, or (5) other. The coding scheme achieved high inter-rater reliability among the two independent raters (Cohen’s κ = 0.98); any discrepancies in coding were resolved by consensus.

Out of the overall sample of 87 participants, 68 (78%) adolescents provided clarifying information regarding the reason for the strength of their interest in genetic nicotine addiction susceptibility testing (see Table 1). There were no differences between participants who did and did not elaborate upon their responses based on age or gender; however, non-Caucasian adolescents were less likely to elaborate than were Caucasian adolescents (62% *vs.* 85%), χ^2^ (1) = 6.00, *p* = 0.014. Most adolescents indicated that the personal information to be gained from genetic testing would be either useful (*n* = 20, 29%) and would directly affect smoking cessation/prevention, or interesting (*n* = 25, 37%) and would indirectly affect smoking cessation/prevention. Fewer adolescents reported that the personal information to be gained from genetic testing would be either irrelevant (*n* = 9, 13%) due to their self-proclaimed nonsmoking status, or otherwise unimportant (*n* = 12, 18%).

### 2.3. Association with Smoking Behavior

Finally, we explored whether or not interest in nicotine addiction susceptibility testing was associated with whether or not the adolescent with ADHD had ever smoked cigarettes. A Student’s *t*-test was used to compare the mean scores of ever smokers (*M* = 2.59, *SD* = 1.07) *vs.* never smokers (*M* = 2.70, *SD* = 1.13) and this result was not significant, *t* (85) = −0.46, *p* = 0.64. Additional independent variables, such as participant age, gender, race, and income, were examined as well. However, the findings were less robust than those presented herein (data not shown).

### 2.4. Summary and Interpretation

This study investigated the extent to which adolescents diagnosed with ADHD would be interested in genetic testing for nicotine addiction susceptibility if it were available and offered to them. It also assessed adolescents’ reasons for their level of interest and other key variables. Results indicated that most adolescents would be interested in nicotine addiction susceptibility testing and that the information to be gained from genetic testing would either directly or indirectly affect adolescents’ smoking cessation/prevention efforts--independent of their cigarette smoking history. Adolescents’ expressed desire to know their risk for nicotine addiction was related to their desire to lead a smoke-free life (e.g., “*If I was at a greater risk for becoming addicted [to cigarettes] I’d go out of my way to avoid the temptation.*”) or curiosity about personal health information (e.g., “*I would want to know and be aware of anything that could happen to me.*”).

The current study’s findings are similar to two previous studies reporting that adolescents in the general population and those coping with a chronic disease were interested in nicotine addiction susceptibility testing [36,45]. The current study is unprecedented in that it assessed interest in genetic testing for nicotine addiction susceptibility among adolescents with ADHD—a population that is at high risk for smoking cigarettes. To our knowledge, few works have assessed genetic testing interest among children and adolescents who were at high risk for a specific health concern (*i.e.*, heart disease or breast cancer) [42]. Furthermore, only one other study investigated the potential impact of genetic testing on specific adolescent health behavior [36]. The distinctive information provided by the current study is that many high risk adolescents with ADHD are interested in genetic susceptibility testing, and believe the results of this genetic test would affect their smoking behavior. This was true regardless of previous smoking behavior, indicating that genetic susceptibility testing may have an impact on both smoking uptake and smoking cessation. The results provide early/preliminary support for continuing research regarding genetic biomarkers for risk of nicotine addiction in high priority populations.

The importance of increasing our knowledge regarding nicotine addiction susceptibility biomarkers cannot be overstated. Research has consistently demonstrated that adolescents with ADHD are more likely to smoke cigarettes than healthy adolescents [9]. Symptoms of ADHD (*i.e.*, impulsivity and inattention) have been proposed to impact the likelihood that an adolescent will try cigarettes and become addicted to nicotine [6,12]. However, there may be additional predisposing factors that have not yet been discovered, such as common genetic etiology for both ADHD and nicotine addiction. Increased information regarding the underlying genetic aspects of these two disorders could potentially provide a greater understanding of the mechanisms leading to both ADHD and nicotine addiction. This may one day translate into genetic tests capable of identifying adolescents with ADHD who are prone to developing smoking habits.

There are potential long-term clinical implications for the identification and use of nicotine addiction susceptibility biomarkers as well. Extensive evidence indicates that pharmacological interventions are a key component of the treatment of ADHD symptoms [46] and individuals diagnosed with ADHD have less success in smoking cessation programs than individuals without ADHD [13]. The identification of addiction susceptibility biomarkers may allow researchers to develop pharmacological treatments that concurrently address both ADHD symptoms and nicotine addiction risk. Furthermore, health care providers may be able to use genetic screening to tailor interventions and optimize care in order to address adolescents’ symptoms from a more comprehensive biopsychosocial approach. Given the staggering number of annual deaths and millions of dollars per year of lost productivity related to tobacco use [3] and the high risk for nicotine addiction among individuals with ADHD, the potential benefits of nicotine addiction-related genetic research are vast.

Despite the possible gains of nicotine addiction susceptibility testing, there are also recognized caveats. As noted earlier, many questions about the relationship between ADHD and nicotine addiction remain unanswered, especially those examining causal mechanisms of action. It would be prudent for researchers to continue to examine this relationship, with an eye toward common genetic and/or environmental etiologies for both traits, both alone and in combination with one another. Furthermore, genetic susceptibility testing is not a panacea for future health concerns. There is scant evidence that providing individuals with information about risk biomarkers reduces smoking behavior, and there may be unforeseen consequences to such testing. For example, individuals who are known to be at normal risk of nicotine addiction may be encouraged to smoke or deterred from seeking cessation programs [47]. Although unlikely, this possibility should be explored in future studies. Alternatively, individuals with a higher risk of smoking may believe that smoking prevention or cessation programs will not be successful because they are “destined” to be addicted to nicotine. Additionally, genetic testing for a disorder is not as clear-cut as it may have once seemed. Many genes are pleiotropic in nature, meaning they influence multiple phenotypic traits. Genes that are associated with the dopamine transporter system, one of several predominant gene systems being studied with respect to nicotine addiction, are related to other mental health conditions as well [48]. Information about nicotine addiction risk or ADHD could reveal information about other potential health outcomes. Finally, there are a variety of ethical considerations as well, such as the stigma that may result from a societal view of nicotine addiction as a medical problem relative to other addictive disorders [49]. Thus, researchers and health care providers need to consider an array of ramifications prior to using genetic screening with clinical populations. Continued research about the consequences of genetic testing is warranted.

## 3. Experimental Section

Demographic characteristics of the 87 participants in this study are provided in Table 2. Participants were 87 healthy adolescents between the ages of 13 and 21 (*M* = 16.64, SD = 2.17) who had been diagnosed with ADHD; 43% of the sample was male. Participants were recruited for this study upon attending a general medical check-up at an adolescent medicine clinic in an urban ambulatory care center. Adolescents were eligible for this study if they were: (1) within the specified age range, (2) able to read and comprehend English, (3) able to provide valid informed consent/assent, (4) in good general health as determined by parental or self-report, and (5) had a confirmed diagnosis of ADHD. ADHD diagnoses were determined by parental report with a structured interview [50], clinician report based on standard psychiatric diagnostic criteria [8], and medical record review. Participants’ current symptoms were confirmed using an ADHD behavior rating scale [50].

Potential participants were screened by research assistants who attended the adolescent medicine clinic at predetermined times. Research assistants directly approached adolescents who were 18 years of age and older. Parents/legal guardians of adolescents who were under 18 years of age were approached by research assistants prior to speaking with the adolescent. Once written consent/assent was obtained, participants completed a self-administered behavioral survey, provided a DNA sample in order for genes hypothesized to relate to smoking behavior to be isolated, and underwent a biochemical verification test of recent cigarette smoking (exhaled carbon monoxide). Participants were informed that they would not receive the results of their genetic test because clinical genetic testing for nicotine addiction susceptibility is not yet available. Participants received a small incentive as an acknowledgement of their time. There was an approximate 88% participation rate in the study, and the majority of adolescents who declined to participate did so due to a reported lack of time.

Demographic variables: age, gender, and race data were collected from each participant. Participants’ home address was used to create an area-based socioeconomic measure (via conversion of zip code to median household income [a mid-point division of income into equal groups]) [51]. This approach is commonly used in behavioral epidemiologic investigations designed to understand multilevel influences on clinical health outcomes (e.g., environmental resources, risk and protective factors) [52].

Smoking status: participants’ smoking status was determined by their response to several standard epidemiological questions regarding experimentation with smoking, number of cigarettes smoked throughout one’s life, and the amount of time that had passed since the participant last smoked [53].

Interest in nicotine addiction susceptibility testing: the dependent variable for this study was participants’ interest in a hypothetical genetic test for nicotine addiction susceptibility. Participants’ responded to the following item on a 4-point Likert scale (1 = Not at all to 4 = A lot): “*Someday, scientists may be able to predict who is at greater risk for growing up to become addicted to smoking, based on a person’s genes (their DNA). If this information were available, how strongly would you be interested in learning about your genetic risk for developing a smoking habit?*” An open-ended question clarifying the rationale of the adolescents’ interest in genetic testing followed the item. These responses were then content-coded by two independent raters using a previously developed coding scheme [36].

## 4. Conclusions

There are several limitations to this study. First, the current investigation’s sample size was modest and affected our ability to conduct additional statistical analyses. Second, the majority of adolescents in this sample were Caucasian and from moderately-resourced families. The extent to which adolescents of other backgrounds might be interested in genetic testing for nicotine addiction susceptibility is not known; further study with more representative samples is also necessary. Third, adolescents who participated in this study consented to provide a DNA sample to identify genes related to nicotine addiction. This may have led to positive bias among participants’ responses to the primary outcome of interest. And finally, a single-item measure was used to assess adolescents’ interest in genetic testing. It is advised that future studies expand on this approach. Despite these limitations, the work provides novel information about a population that is well-documented to be at increased risk for nicotine addiction. These preliminary data may lead to further research regarding adolescents with ADHD, nicotine addiction, and genetic testing.

Over the last decade, interest in, and applications for genetic testing have expanded greatly [54]. The human genome can now be sequenced and specific markers for future health disorders are rapidly being identified. The practice of medicine is increasingly becoming personalized, with greater emphasis on risk prediction and targeted and tailored interventions [55,56]. The present study provides preliminary evidence that adolescents with ADHD, a high risk population for developing nicotine addiction, are interested in genetic screening if available, suggesting that it would be prudent for researchers to continue researching genetic biomarkers for nicotine addiction susceptibility and ADHD. This research has the potential to provide novel information about the relationship of ADHD and nicotine addiction and may lead to better identification of adolescents who are at high risk for smoking and enhanced smoking prevention/cessation programs. Presently, it will be important for researchers, health professionals, and public health specialists to consider both the positive and negative ramifications of genetic testing and proceed with caution. Future studies regarding the ethical implications of genetic testing should be conducted prior to incorporating this approach into health care interventions.

## Figures and Tables

**Table 1.** Adolescents’ elaborated reasons behind the strength of their interest in nicotine addiction susceptibility testing (*n* = 68).

**Reason**	**Description**	***n***	**%**
Interesting	Indication of a general/nonspecific interest in, or curiosity about, personal information that would be learned as a result of participation in testing without any expressed behavioral intent (e.g., altruistic, ‘a test/result is good to know for the future,’ ‘curious for future generations’)	25	37
Useful	Indication of a specific interest in, or curiosity about, personal information that would be learned as a result of participation in testing with the expressed intent of preventing smoking behavior or stopping smoking (e.g., risk avoidance, active maintenance of current/future health)	20	29
Irrelevant	Indication that personal information that would be learned as a result of participation in testing is irrelevant due to self-proclaimed nonsmoking status (e.g., ‘a test/result doesn’t matter because I will never smoke’)	9	13
Unimportant	Indication that personal information that would be learned as a result of participation in testing is generally unimportant, uninteresting, or insignificant (e.g., ‘a test/result would not affect my decisions,’ ‘it is not useful’)	12	18
Other	Miscellaneous (e.g., references to familial cancer, ‘just because,’ other inexplicable responses)	2	3

**Table 2.** Demographic characteristics (*n* = 87).

**Variable/level**	**Mean (*SD*) or *n* (%)**
Age, in years	16.64 (2.17)
Gender	
Male	37 (43)
Female	50 (57)
Race	
Caucasian	61 (70)
African American	14 (16)
Hispanic	7 (8)
Other	5 (6)
Income, in $	72,452 (23,404)
Lifetime smoking	
No	41 (47)
Yes	46 (53)
